# Finite-element analysis of the proximal tibial sclerotic bone and different alignment in total knee arthroplasty

**DOI:** 10.1186/s12891-019-3008-z

**Published:** 2019-12-26

**Authors:** Ye-Ran Li, Yu-Hang Gao, Chen Yang, Lu Ding, Xuebo Zhang, Hanzhe Chen, Jianguo Liu, Xin Qi

**Affiliations:** Department of Orthopaedic Surgery, The First Hospital of Jilin University, Jilin University, Xinmin St 71, Chang Chun, China

**Keywords:** Mechanical alignment, Kinematic alignment, Contact stress, Finite element model, Computer simulation

## Abstract

**Background:**

Despite potential for improving patient outcomes, studies using three-dimensional measurements to quantify proximal tibial sclerotic bone and its effects on prosthesis stability after total knee arthroplasty (TKA) are lacking. Therefore, this study aimed to determine: (1) the distribution range of tibial sclerotic bone in patients with severe genu varum using three-dimensional measurements, (2) the effect of the proximal tibial sclerotic bone thickness on prosthesis stability according to finite-element modelling of TKA with kinematic alignment (KA), mechanical alignment (MA), and 3° valgus alignment, and (3) the effect of short extension stem augment utilization on prosthesis stability.

**Methods:**

The sclerotic bone in the medial tibial plateau of 116 patients with severe genu varum was measured and classified according to its position and thickness. Based on these cases, finite-element models were established to simulate 3 different tibial cut alignments with 4 different thicknesses of the sclerotic bone to measure the stress distribution of the tibia and tibial prosthesis, the relative micromotion beneath the stem, and the influence of the short extension stem on stability.

**Results:**

The distribution range of proximal tibial sclerotic bone was at the anteromedial tibial plateau. The models were divided into four types according to the thickness of the sclerotic bone: 15 mm, 10 mm, 5 mm, and 0 mm. The relative micromotion under maximum stress was smallest after MA with no sclerotic bone (3241 μm) and largest after KA with 15 mm sclerotic bone (4467 μm). Relative micromotion was largest with KA and smallest with MA in sclerotic models with the same thickness. Relative micromotion increased as thickness of the sclerotic bone increased with KA and MA (R = 0.937, *P* = 0.03 and R = 0.756, *P* = 0.07, respectively). Relative micromotion decreased with short extension stem augment in the KA model when there was proximal tibial sclerotic bone.

**Conclusions:**

The influence of proximal tibial sclerotic bone on prosthesis’s stability is significant, especially with KA tibial cut. Tibial component’s short extension stem augment can improve stability.

## Background

Osteosclerosis usually occurs below the medial tibial plateau of patients with osteoarthritis (OA) exhibiting severe genu varum before surgery. Ishii found that the bone density was significantly higher below the medial tibial plateau than at other tibial plateau sites in these patients [1]. By examining plain films, other studies have found that proximal tibial sclerotic bone lesions are often adjacent to the cortical bone-cartilage interface [2–4]. There are many studies focused on the microstructure of the sclerotic bone [5–7], however, this study makes a novel contribution to the literature by using three-dimensional (3-D) measurements to quantify the location and scope of proximal tibial sclerotic bone and finite-element modelling to describe the effect of the proximal tibial sclerotic bone on the stability of the tibial prosthesis.

Currently, there are two tibial cut methods used in clinical practice during total knee arthroplasty (TKA). The mechanically aligned (MA) technique places the tibial prosthesis perpendicular to the tibial mechanical axis. This keeps the force on the medial and lateral sides of the tibial plateau consistent, thus improving the stability of the prosthesis. In kinematic alignment (KA), the natural joint line of the knee is restored to improve functional recovery after surgery [8, 9]. Several studies have reported on the effects of KA and MA tibial cut on the stability of the tibial prosthesis of patients with severe genu varum of the knee [10–13]. However, there is a gap in knowledge on the effect of proximal tibial sclerotic bone on the stability of the prosthesis with tibial cut performed at different angles in patients with severe preoperative genu varum. In addition, Park found that when genu varum is greater than 8° preoperatively, the use of a short extension stem augment under the tibial component can effectively reduce the loosening rate of the tibial prosthesis [14]. However, when there is proximal tibial sclerotic bone, the effectiveness of the short extension stem augment is unknown.

Therefore, this study aimed to determine: (1) the approximate distribution range of the proximal tibial sclerotic bone by 3-D reconstruction, (2) the effect of the proximal tibial sclerotic bone on the stability of the tibial prosthesis in TKA with 3 different alignments and simulated surgery using finite-element analysis of patients with severe genu varum due to OA, and (3) the effect of a an extension stem on the stability of a prosthesis after KA.

## Methods

### Three-dimensional proximal tibial sclerotic bone models

**Fig. 1:**
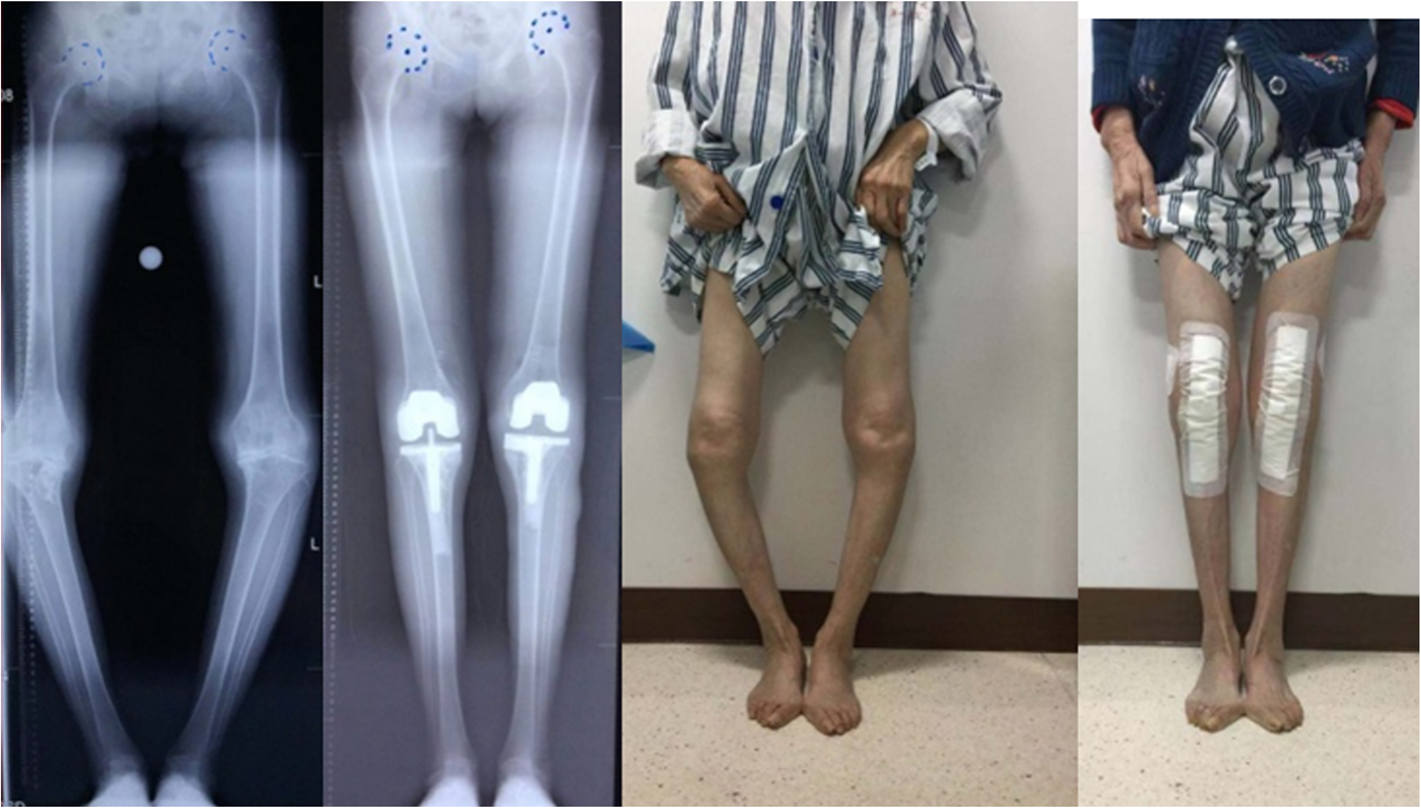
Clinical images for a severe varus OA case preoperatively and postoperatively

**Fig. 2:**
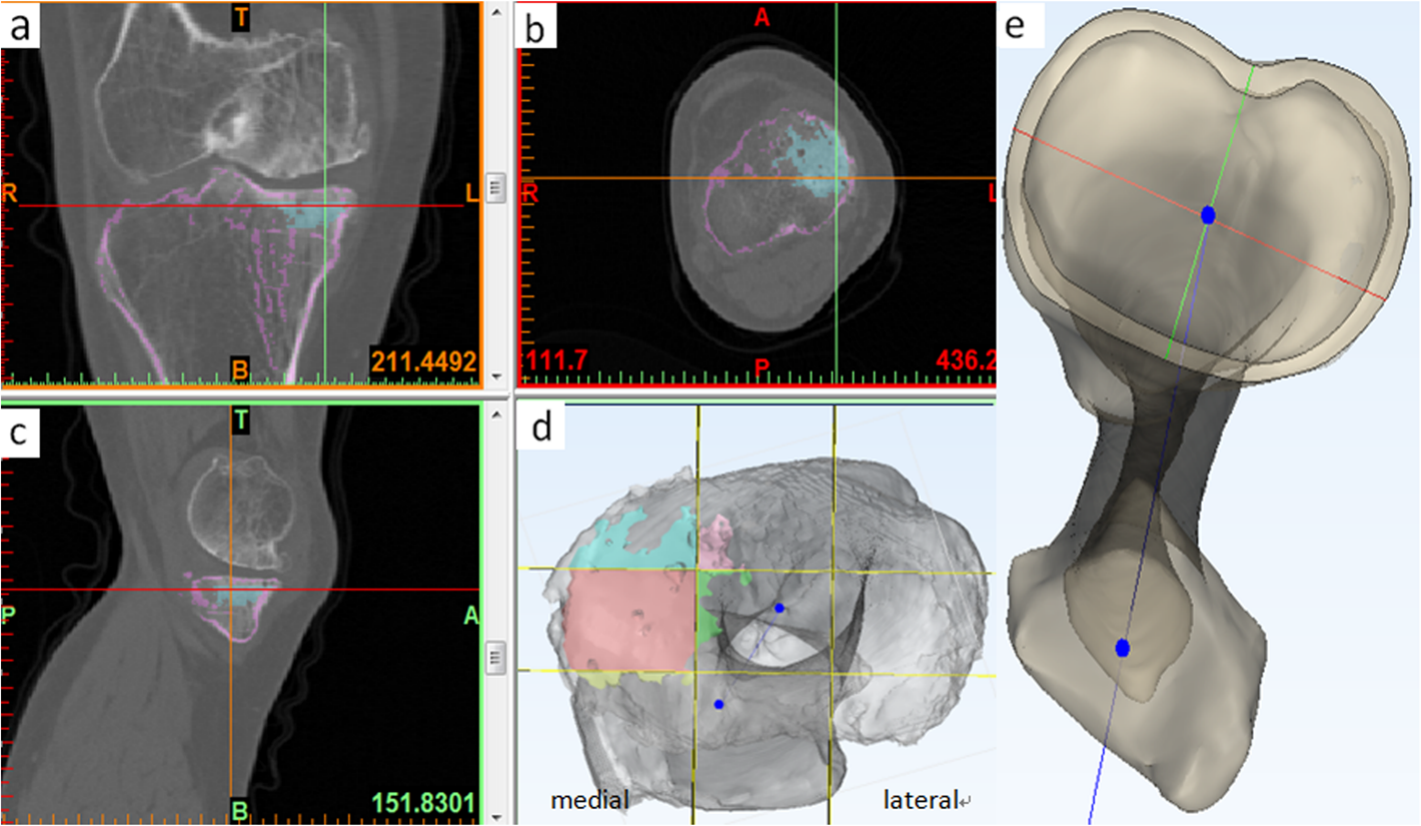
The proximal tibial sclerotic bone area of a severe right varum genu from CT images and the distribution obtained by the 3D reconstruction technique. T: top; B: bottom; L: left (medial); R: right (lateral); A: anterior; P: posterior. Blue line: Z axis (Mechanical alignment,); Green line: Y axis(AP axis); Red line: X axis

From January 2018 to January 2019, patients with knee OA and severe genu varum over 15° were selected for 0.6 mm thin-layer CT scanning of both lower extremities and 3-D modelling using Mimics 14.0 (Materialize, Leuven, Belgium) A severe varus OA case’s clinical images were shown in Fig. 1. Patients with rheumatoid arthritis, suspected preoperative infection, or who previously underwent knee surgery were excluded from the study. The following coordinate system was established for the modelling, as shown in Fig. 2: the z-axis was the tibial MA, which was defined as the line connecting the center of the ankle joint and the center of the random axial levels below the articular surface of the proximal tibia and above the tibial tuberosity; the y-axis was defined as the anterior-posterior (AP) axis, which was the connection between the medial edge of the tibial tuberosity and the midpoint of the posterior cruciate ligament after an experienced orthopedist recommended omitting the effect of the osteophyte in the plane perpendicular to the z-axis; the line perpendicular to the y-z axis was defined as the x-axis, and the fibula was disregarded to simplify the model (Fig. 2e). The tibial cut was performed on the plane perpendicular to the tibial MA and 8 mm below the lateral tibial plateau surface. Cases were included in the study if there were no defects in the medial tibia. If there was a defect in the medial tibia less than 4 mm from the tibial cut plane, the amount of lateral tibial cut was increased until the defect was removed, and the case was included. If the medial defect was greater than 4 mm, the case was excluded. Among those 120 cases, 4 cases were excluded from the study based on a preoperative posterior drawer test which was strongly positive (+++) and subsequent diagnosis of posterior cruciate ligament injury. According to these criteria, a total of 116 patients were included in the study. Basic statistical data are shown in Table 1.

**Table 1:** Baseline characteristics among 3 groups

Thickness of Subchondral sclerosis	< 5 mm	5–10 mm	> 10 mm	*P* value
Gender (m/fm)	*10/34*	8/30	8/26	1.00
Age(years)	65 ± 7.01	66 ± 6.89	67 ± 7.1	0.78
Height(cm)	160 ± 6.59	160 ± 6.69	160 ± 6.16	0.90
Weight (kg)	65 ± 9.05	65 ± 9.03	65 ± 9.01	0.62
BMI (kg/cm^2^) Average volume of sclerotic bone (mm^3^)	26 ± 3.7 808	26 ± 3.59 2444	26 ± 3.2 4016	0.95

Continuous data were presented as mean ± SD. A significant difference between groups was considered for *p* < 0.05

Within the sample population, the proximal tibial sclerotic bone area was defined as the area with a Hounsfield unit (HU) value of 400–2000 HU in the CT image (based on Mimics software default classification of: bone = 220–2000 HU, compact bone = 660–2000 HU, and spongy bone = 148–661 HU). After auto-selection by Mimics software, the experienced orthopedists and imaging doctors rescreened areas with abnormal HU values from the original cancellous bone after a simulated tibial cut, establishing a 3-D tibial sclerotic bone model (Fig. 2a-c). Since zoning methods such as the whole-organ magnetic resonance imaging score (WORMS) and the magnetic imaging OA knee score (MOAKS) are based on the coronal, sagittal and axial views from magnetic resonance imaging, and the quantitative partitioning could not be accurately performed, we optimized WORMS and MOAKS methods to improve the accuracy of the quantitative partition of the knee on the tibial side [15, 16], as shown in Fig. 2d. For this, a plane perpendicular to the y-axis and another plane parallel to that plane were created. The proximal tibia was equally divided into three portions by the two planes in the x-axis direction. Two planes perpendicular to the existing two planes parallel to the y-axis were created and the two planes were extended toward the y-axis to divide the tibia into three portions equally (Fig. 2d). According to this method, the proximal tibia was divided into the inner anterior region, the inner central region, and the inner posterior region. The sclerotic bone was also divided according to the modified proximal tibial partition. The total volume, sclerotic bone volume in each zone, the relative volume ratio, and the thickness between the tibial cut plane and the distal end of the sclerotic bone were recorded. According to the thickness of sclerotic bone, the models were divided into four types: large (L) = 15 mm, medium (M) = 10 mm, small (S) = 5 mm, and none (N) = 0 mm. The axial distribution of the sclerotic bone models was set according to the mean value of the 3-D measurement distribution ratio. Based on the four types of sclerotic bone models, the different tibial cut angles were categorized. The MA tibial cut was perpendicular to the tibial alignment, and the KA and valgus 3° tibial cut were simulated by 3° varus and valgus rotation of the previous MA tibial cut plane on the x-z coordinate system. Based on the 4 types of tibia and proximal tibial sclerotic bone tibial cut models and 3 groups of alignments, the sclerotic bone models comprised the following 12 variations: L-MA, L-KA, L-valgus 3°; M-MA, M-KA, M-valgus 3 °; S-MA, S-KA, S-valgus 3°; N-MA, N-KA, N-valgus 3°.

### Modelling the effect of proximal tibial sclerosis on the tibial prosthesis in total knee arthroplasty

In order to produce a basic model without any sclerotic bone, a 45-year-old female volunteer with healthy knee joints was selected to undergo a 0.6 mm thin-layer CT scan.

The cortical and cancellous bones were modelled using Mimics software and the coordinate system and alignments were established as described above. First, MA tibial cut was simulated where the cortical and cancellous bone were osteotomized 8 mm from the lateral tibial surface perpendicular to the tibial alignment. The basic model was obtained after checking its integrity.

To determine the tibial components, the appropriate tibial plateau model was selected, and the model profile was obtained by a direct measurement according to the Press-Fit Condylar Sigma system (DePuy Orthopaedics, Warsaw, Ind) and assembled according to the manufacturer’s guidelines. The tibial plateau rotation and the partition method shared the same y-axis, which was the tibia AP axis, and an 8-mm spacer was chosen. Since a motion model was not used, the profile above the spacer could be omitted and replaced with a platform [17, 18]. We neglected the element of bone cement as in previous research models [12, 19, 20].

**Fig. 3:**
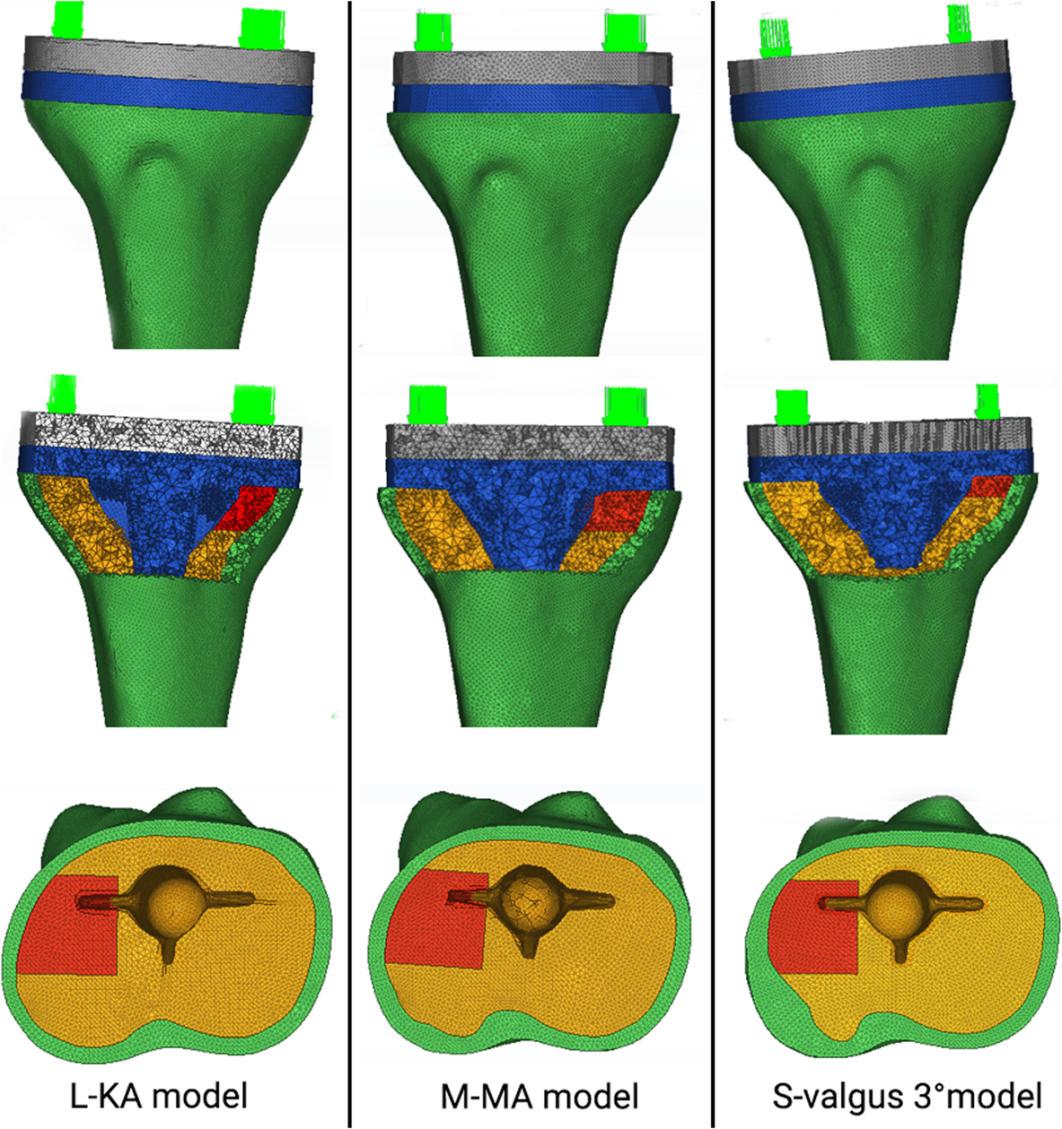
Finite element analysis of the L-KA model, M-MA model, and S-valgus 3°model. Coronal, coronal section and axial view of the finite element model. S: small = 5 mm; M: medial =10 mm; L: large =15 mm; MA: mechanical alignment; KA: kinematic alignment

The finite element model of sclerotic bone was created according to the 3 groups of 4 types established in Part 1 and the tibial plateau and the spacer model were installed. After confirming that the 3-D model was completely free of overlapping and no deformity, HyperMesh (Altair HyperWorks, Troy, MI, USA) was used for finite-element meshing. Check the quality of finite element mesh to ensure no overlap or deformation established. Figure 3 shows the L-KA, M-MA, and S-valgus 3° finite-element models. The prosthetic stem was increased by 30 mm using the same prosthesis to simulate an extended stem prosthesis. The L-KA, M-KA, S-KA, and N-KA models were made in the same way and finite-element meshing was performed using HyperMesh software.

### Material properties, load, and boundary conditions

According to previous research data, the tibial cortex was set at E = 17,000 MPa with Poisson’s ratio 0.3, medullae at E = 400 MPa and Poisson’s ratio 0.3, tibial plateau at E = 248,000 MPa with Poisson’s ratio 0.3, spacer at E = 667 MPa with Poisson’s ratio 0.46, sclerotic bone set at E = 1726 MPa with Poisson’s ratio 0.3 [18, 21–24]. Contact between the tibia model and tibial plateau were treated as a frictional contact problem with a friction coefficient of 0.2 [25]. In order to ensure the convergence of displacement and stress, the mesh size of the stress region and contact region in the model was refined to the minimum of 1 mm, and the mesh independency was checked. The convergence tolerance of the contact analysis was set to 1%, similar to Abdul-Kadir et al. (2008), which provided an accurate solution to the contact analysis of joint replacement [26].

The inferior surface of the distal tibia was fixed in all directions and the maximum load and the medial and lateral distributions were similar to those in previous studies [17, 21, 22, 27, 28]. In particular, simulations were set at: KA tibial cut with 65% stress on the medial side and 35% stress on the lateral side of the tibia, MA tibial cut with 50% stress on the medial side and 50% stress on the lateral side of the tibia, and tibial cut of valgus 3° with 35% stress on the medial side and 65% stress on the lateral side of the tibia [27]. The force was set at three times of the total body weight (e.g. 70 kg*3 = 2100 N). The force-bearing site was the transverse diameter of the tibial prosthesis across the medial and lateral plateau center [17, 21, 22, 28] and was vertical on the upper surface of the spacer. The relative micromotion displacement beneath the stem and the force distribution of the prosthesis and the tibia were compared [18].

### Statistical analysis

All data are expressed as means ± standard deviations. Pearson’s correlation coefficient is provided to assess the extent of linear association relative micromotion beneath the stem in 4 groups(KA, MA, 3° valgus and KA with short extension stem augment). Statistical significance was defined as *P* < 0.1. Statistical analyses were performed using SPSS 17.0 (SPSS, Chicago, IL).

## Results

After simulated tibial cut on the 116 cases selected for the study, the region of sclerotic bone in these cases were described according to the modified WORMS. The mean thickness of the remaining 116 sclerotic bone cases was 7.13 mm (range, 5.25 to 7.26 mm) and mean volume was 2155 mm^3^ (range, 1633 to 2011 mm^3^). The average volume of sclerotic bone in the anterior region accounted for 28% of the total volume, the central region accounted for 61%, and the posterior region accounted for 7%. Accordingly, when the proximal tibial plateau was divided into 9 equal parts, the sclerotic bone of the proximal tibia in the severe varum knee was generally located in the area of 3/9 to 5/9 in the medial plateau. The 116 models were classified according to the thickness of sclerotic bone in the proximal tibia after the osteotomies. Among which, 44 cases were less than 5 mm, and the average volume of sclerotic bone was 808 mm^3^; 38 cases were greater than or equal to 5–10 mm, and the average volume of sclerotic bone was 2444 mm^3^; 34 cases were greater than 10 mm, of which 8 cases were thicker than 15 mm, and the average volume of sclerotic bone was 4016 mm^3^. There was no difference in basal value among the three groups (Table 2). According to the thickness of sclerotic bone, the models were divided into four types: Large 15 mm (L), medium 10 mm(M), small 5 mm(S), and none 0 mm(N) respectively.

**Table 2:** Material properties used in the FE model

Part of FE Model	Elastic Modulus (MPa)	Material Property Poisson’s Ratio	Density (g/cm^3^)
Cortical Bone	17,000	0.3	1.64
Cancellous Bone	400	0.3	0.27
Sclerotic Bone	1726	0.3	1.054
Tibial Spacer	667	0.46	8
Tibial Baseplate	248,000	0.3	0.94

**Fig. 4:**
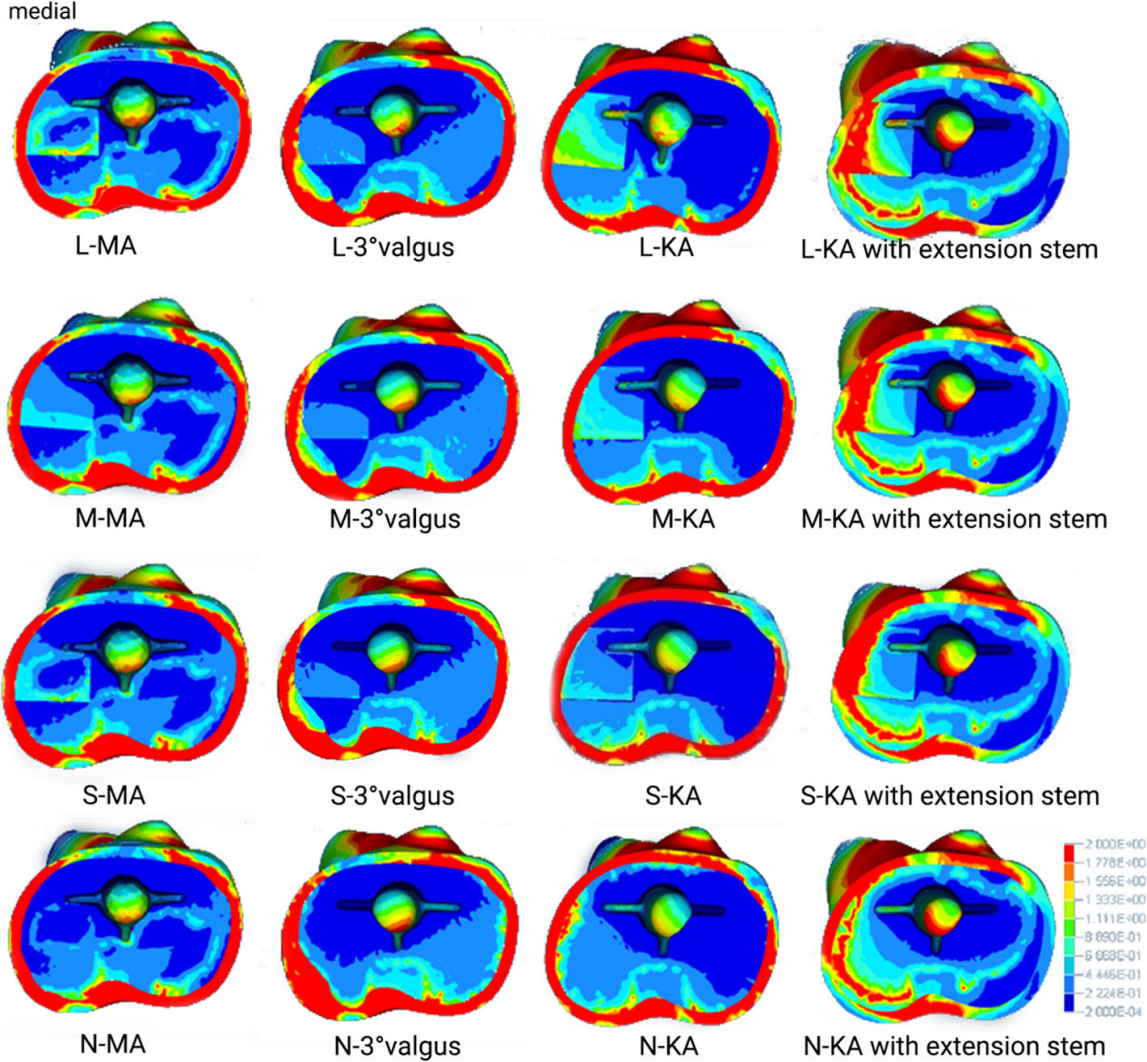
Top view of von Mises stress distribution on the contact surface of the tibia under maximum loading in each finite element model. N: none = 0 mm; S: small = 5 mm; M: medial = 10 mm; L = 15 mm; MA: mechanical alignment; KA: kinematic alignment. Left side on the figure indicates medial condyles

Figure 4 shows the stress distribution in the proximal tibia bone. With increased thickness of the sclerotic bone, the stress of the sclerotic bone area increased gradually in all tibial cut alignment groups; stress concentration of the cancellous bone near the posterolateral stem was discovered in the MA group and valgus tibial cut group while in the KA group, the stress concentration of the cancellous bone near the posteromedial stem was observed.

**Fig. 5:**
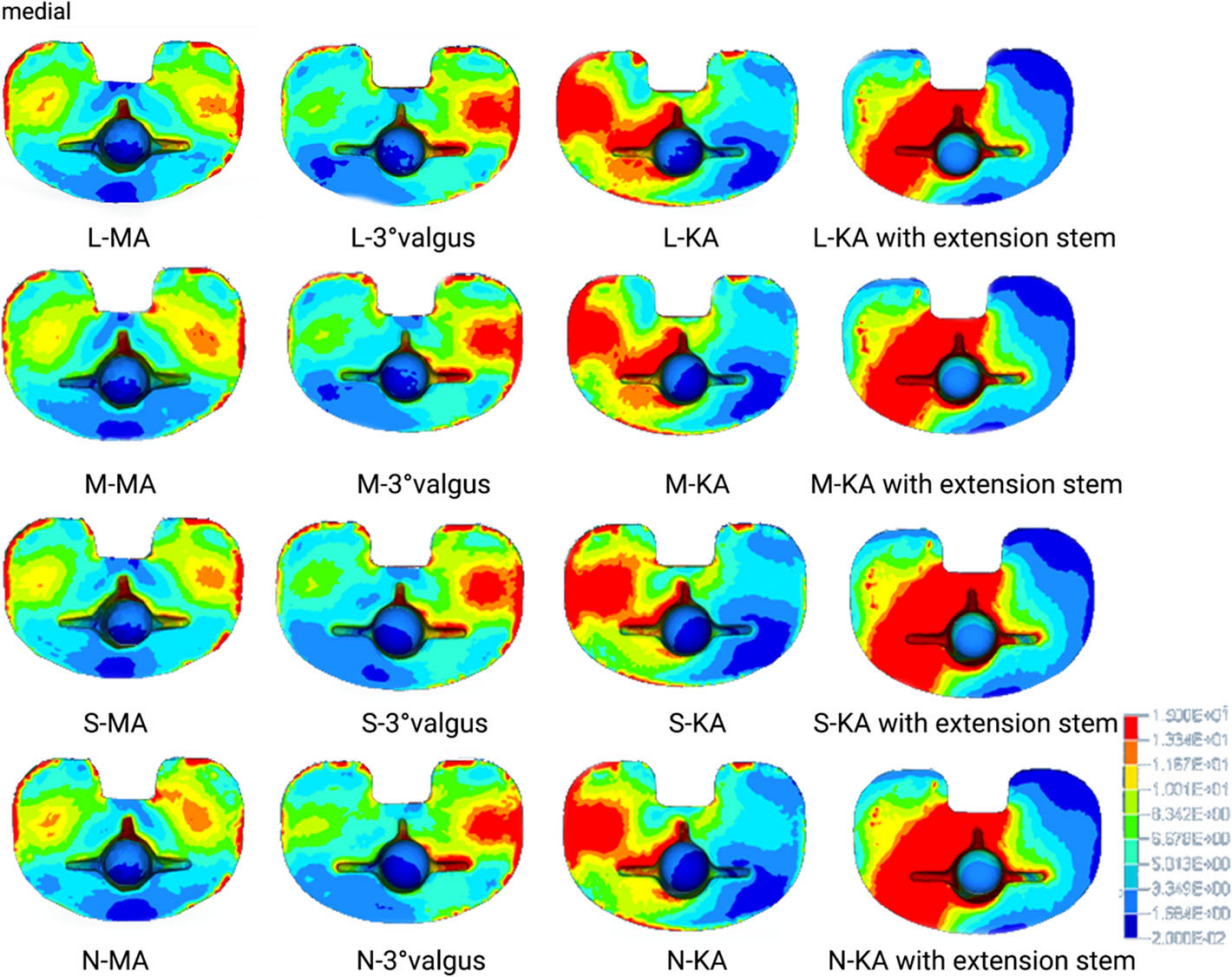
Bottom view of von Mises stress distribution on the contact surface of the tibial prothesis under maximum loading in each finite element model. N: none = 0 mm; S: small = 5 mm; M: medial = 10 mm; L = 15 mm; MA: mechanical alignment; KA: kinematic alignment. Left side on the figure indicates medial condyles

Figure 5 shows the stress distribution of the tibial prosthesis. With the increase in thickness of sclerotic bone, the stress distribution of the medial plateau and medial keel was more concentrated in the KA group, the stress distribution of the medial plateau was also concentrated in the MA group, and stress concentration in the lateral plateau was detected in the valgus tibial cut group.

**Fig. 6:**
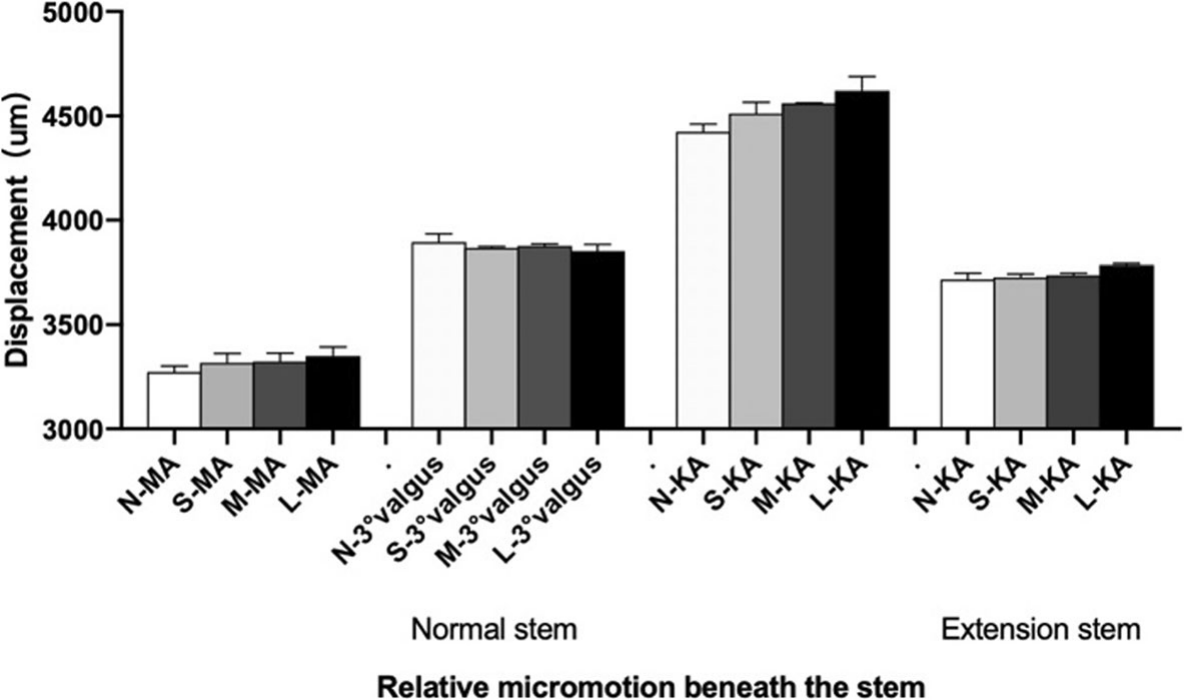
The relative micromotion displacement of the distal end of the prosthesis in each model. N: none = 0 mm; S: small = 5 mm; M: medial =10 mm; L: large =15 mm; MA: mechanical alignment; KA: kinematic alignment

Figure 6 shows the comparison of the relative micromotion displacement beneath the stems among different groups. In the non-sclerotic bone group and models with the same thickness, the relative micromotion displacement beneath the stem was the largest when KA tibial cut was performed (20% higher than that in the MA group on average and 9% higher than that in the valgus group), indicating that the KA tibial cut prosthesis was the most unstable under the same thickness of the sclerotic bone. With the increase of sclerotic bone thickness, the relative micromotion displacement beneath the stem increased in each KA and MA tibial cut group (R = 0.756, *P* = 0.07 in the MA group, R = 0.937, *P* = 0.03 in the KA group, where *p* < 0.1 was considered as statistically significant). In the valgus tibial cut group, the relative micromotion displacement beneath the stem decreased gradually with the increase of the thickness of the sclerotic bone but the difference was not significant (R = -0.867, *P* = 0.133, where p < 0.1 was considered as statistically significant).

When the prosthesis stem that was placed on simulated KA tibial cut was lengthened by 30 mm, the stress of any thickness sclerotic model in the medial tibial plateau and short extension stem augment increased significantly. The stress of the proximal tibial cortex under the prosthesis decreased, and the stress of the sclerotic bone area increased with thickness (Figs. 5 and 6). The relative micromotion displacement beneath the stem was considerably lower than that of the normal length prosthesis, where the average displacement of the KA group was about 12% lower than that of the KA group using the short extension stem augment (Fig. 6).

## Discussion

Our study found that the KA tibial plateau was less stable than the MA and valgus tibial cut when there was no sclerosis in the medial tibia. Previous studies [13, 21, 29, 30] have shown that the tibial plateau had the worst stability in the absence of sclerosis after 2.5° varus tibial cut [30] compared to 3°varus tibial cut [12, 13]. This is consistent with the results of our study comparing the prosthesis stability of the three groups without sclerosis. Nakamura et al. focused on the comparison of prosthesis stability between KA and MA techniques in patients with genu varum.^15^ The study showed that the stress exerted on the bone under the medial plateau was greater in the KA model than in the MA model and the more severe the genu varum preoperatively, the greater the difference between the two models. Although the study was limited to varus tibial cut without interference from proximal and medial tibial sclerosis, the findings are consistent with the results of the present study on the stress distribution of the bones under the varus tibial cut plateau in the absence of sclerosis.

Our study shows that the stability of the prosthesis in KA and MA tibial cut decreases as the thickness of the medial tibial sclerosis increases. In the valgus group, the stability of the prosthesis did not change significantly with increased sclerotic bone thickness. In KA tibial cut, when the prosthesis rod was lengthened by 30 mm, the stability of the prosthesis was improved. In a study by Park et al., when preoperative genu varum > 8°, using a short-length extension stem reduced the tibial prothesis loosening rate post-operatively [14]. However, that study only focused on pre-operative varus and lacked analysis of varus tibial cut. Quilez et al. researched different types of short extension stem augments used during tibial cut. Results showed that the short extension stem augment could share the stress around the cortical bone which was consistent with our results [31]. These results support our findings that the short extension stem augment in KA tibial cut can effectively increase the stability of the prosthesis.

To date, this is the first study to analyze the distribution of sclerotic bone in OA patients with severe genu varum. A study by Cox et al. reported on subchondral sclerosis of the tibia with samples selected from the medial central area of the tibial plateau to determine the cause of the sclerosis in the proximal tibia but did not describe the distribution of sclerotic bone [5]. Despite these strengths, there are few limitations of the present study to consider. Firstly, our model is static and did not add dynamic analysis. In previous studies, the peak force of knee joint appeared in standing position, shortly before contralateral heel-down. Therefore, our model was sufficient for simulating the conditions during peak force on the prosthesis. Secondly, our model does not take the influence of soft tissue and bone cement on the prosthesis into account, but this was similar to other studies [17, 18, 28, 32, 33], due to the fact that bone cement is rigid and has little influence on the process of force transmission. Thirdly, our finite element models were based on approximate simulation of data assignment, so our models were approximations which need to be verified by follow-up results or mechanical model of cadaver. At present, 116 patients were followed up for a maximum of 20 months after the surgery. Therefore, our findings are not conclusive and our methods need to be verified and supported by long-term follow-up results.

## Conclusions

The influence of proximal tibial sclerotic bone on the stability of tibial prosthesis is significant, especially with a KA tibial cut. The risk of the instability of the tibial prothesis increased as the thickness of the sclerotic bone increased with the KA tibial cut. In these cases, a short extension stem augment can improve stability.

## Data Availability

The datasets generated and/or analysed during the current study are not publicly available due to individual privacy but are available from the corresponding author on reasonable request.

## Abbreviations

AP: anterior-posterior
CT: computed tomography
HU: Hounsfield unit
KA: kinematic alignment
L: large
M: medium
MA: mechanical alignment
MOAKS: magnetic imaging osteoarthritis knee score
N: none
OA: osteoarthritis
S: small
TKA: total knee arthroplasty
WORMS: whole-organ magnetic resonance imaging score
